# Female showed favorable left ventricle hypertrophy regression during post‐TAVR follow‐up

**DOI:** 10.1002/kjm2.12808

**Published:** 2024-02-08

**Authors:** Cheng‐An Chiu, Pin‐Rong Chen, Yu‐Ju Li, Chong‐Chao Hsieh, Hui‐Chen Yu, Chaw‐Chi Chiu, Jiann‐Woei Huang, Chun‐Yuan Chu, Tsung‐Hsien Lin, Hsiang‐Chun Lee

**Affiliations:** ^1^ Division of Cardiology, Department of Internal Medicine Kaohsiung Medical University Hospital, Kaohsiung Medical University Kaohsiung Taiwan; ^2^ School of Medicine, College of Medicine Kaohsiung Medical University Kaohsiung Taiwan; ^3^ Division of Cardiovascular Surgery and Department of Surgery Kaohsiung Medical University Hospital Kaohsiung Taiwan; ^4^ Department of Internal Medicine School of Medicine, College of Medicine, Kaohsiung Medical University Kaohsiung Taiwan; ^5^ Lipid Science and Aging Research Center, College of Medicine, Kaohsiung Medical University Kaohsiung Taiwan; ^6^ Institute/Center of Medical Science and Technology, National Sun Yat‐sen University Kaohsiung Taiwan; ^7^ Graduate Institute of Animal Vaccine Technology, National Pingtung University of Science and Technology Pingtung Taiwan

**Keywords:** aortic stenosis (AS), echocardiography, sex differences, transcatheter aortic valve replacement (TAVR)

## Abstract

Transcatheter aortic valve replacement (TAVR) is a well‐established procedure using a catheter‐introduced valve prosthesis for patients with severe aortic stenosis (AS). This retrospective study investigated sex‐related differences in pre‐ and post‐TAVR clinical and hemodynamic outcomes and analyzed data of the first 100 cases at Kaohsiung Medical University Chung‐Ho Memorial Hospital (KMUH) between December 2013 and December 2021. Baseline characteristics, procedural outcomes, mortality rates, and echocardiographic parameters were analyzed and compared between sexes. Among the 100 patients, male (46%) and female (54%) were of similar age (mean age, male 86.0 years vs. female 84.5 years) and of the same severity of AS (mean pressure gradient, male 47.5 mmHg vs. female 45.7 mmHg) at the time receiving the TAVR procedure. Women had smaller aortic valve areas calculated by continuity equation (0.8 ± 0.3 cm^2^ vs. 0.7 ± 0.2 cm^2^, *p* < 0.001). In addition, women had better left ventricle ejection fraction (59.6 ± 14.0% vs. men 54.7 ± 17.2%, *p* < 0.01). In the post‐TAVR follow‐up, regression of left ventricle mass and dimension was better in women than in men. None of the patient died within 30 days after the procedure, and women tended to have a more favorable survival than men (2‐year mortality and overall mortality rate in 8.3 year, women 9.1% and 22.2% vs. men 22.2% and 34.8%; *p* = 0.6385 and 0.1277, respectively). In conclusion, the sex‐based difference in post‐TAVR regression of LV remodeling suggests a need for sex‐based evaluation for patients with severe AS and their post TAVR follow‐up.

## 1 INTRODUCTION

Aortic stenosis (AS), in addition to degenerative mitral valve regurgitation, is the most common acquired valvular heart disease in developed countries, most of which is processed in degenerative progress.^1^ The prevalence of AS is increasing in currently aging populations worldwide.^2^ Over the last two decades, TAVR has become a guideline‐recommended option for patients with degenerative AS, especially for those with relatively high risks for conventional surgical aortic valve replacement (SAVR).^3^, ^4^ TAVR, as compared with medical treatment, significantly reduced mortality from any cause, the composite end point of death from any cause or repeat hospitalization, and cardiac symptoms, despite the higher incidence of major strokes and major vascular events.^3^

With accumulating experience on TAVR and outcomes reports, emerging evidence has shown sex differences in procedural adverse events and women are more commonly having vascular complications.^5^ Thirty‐day stroke and mortality rates are similar between men and women in a recent Asian report.^6^ A study demonstrated that the mortality of both sexes is associated with frailty, whereas females with pulmonary hypertension exhibit increased mortality rate comparing to men.^7^

In Taiwan, TAVR was introduced in 2010 and has become a procedure included in the National Health Insurance reimbursement. At our institute, TAVR has been performed successfully in more than 100 patients, and the follow‐up duration has reached over 8 years. We hypothesized a sex difference in cardiac remodeling in patients who underwent TAVR. To better understand sex differences in pre‐ and post‐TAVR cardiac remodeling and outcomes, this study retrospectively collected clinical and echocardiographic data from 100 consecutive patients who had received successful TAVR at our medical center.

## 2 METHODS

### 2.1 Patients in the study

Patients with symptomatic severe AS who underwent TAVR at the Kaohsiung Medical University Chung‐Ho Memorial Hospital (KMUH) between December 2013 and December 2021 were enrolled in this retrospective study. A total of 100 patients were included in this study without exclusion criteria. The decisions for TAVR involving valve choice and sizing were based on detailed imaging evaluation, multidisciplinary planning, and shared decision‐making with the patients. Data collection was performed at baseline, the index procedure, discharge, and post‐TAVR follow‐up for all subjects. Data management was approved by and conducted under supervision by the KMUH Institutional Review Board (IRB) (KMUHIRB‐E(I)‐20220211). All subjects provided written informed consent for the procedure and the post‐TAVR follow‐up. Periodic echocardiographic assessments were generally performed every 1–12 months, depending on the patient's need, with the recording of clinical status and events.

### 2.2 Study endpoints

The primary endpoint was all‐cause mortality post‐TAVR, which was analyzed by year. The secondary endpoints were major adverse cardiovascular or cerebrovascular events, cardiac or vascular surgery, bleeding or stroke during follow‐up, and the NYHA functional class. Secondary efficacy endpoints were success rate and complications according to the Valve Academic Research Consortium (VARC)‐3 criteria^8^.

### 2.3 Echocardiography

**FIGURE 1:**
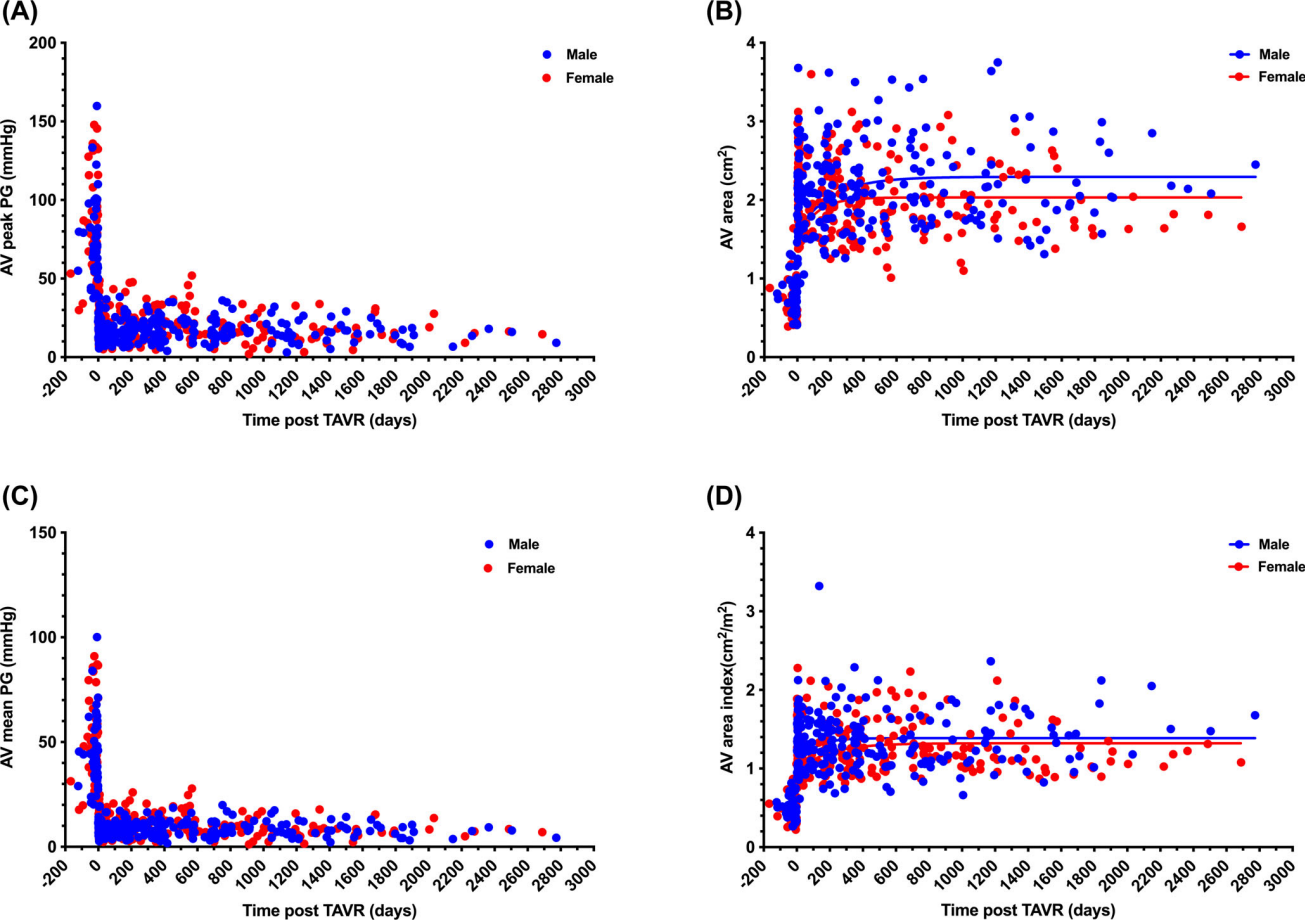
Changes in aortic valve pressure gradient and estimated aortic valve area after transcatheter aortic valve replacement. All the calculated values are shown as scattered points with nonlinear regression curves. Blue points and lines for males; red points and lines for females. AV, aortic valve; PG, pressure gradient.

**FIGURE 2:**
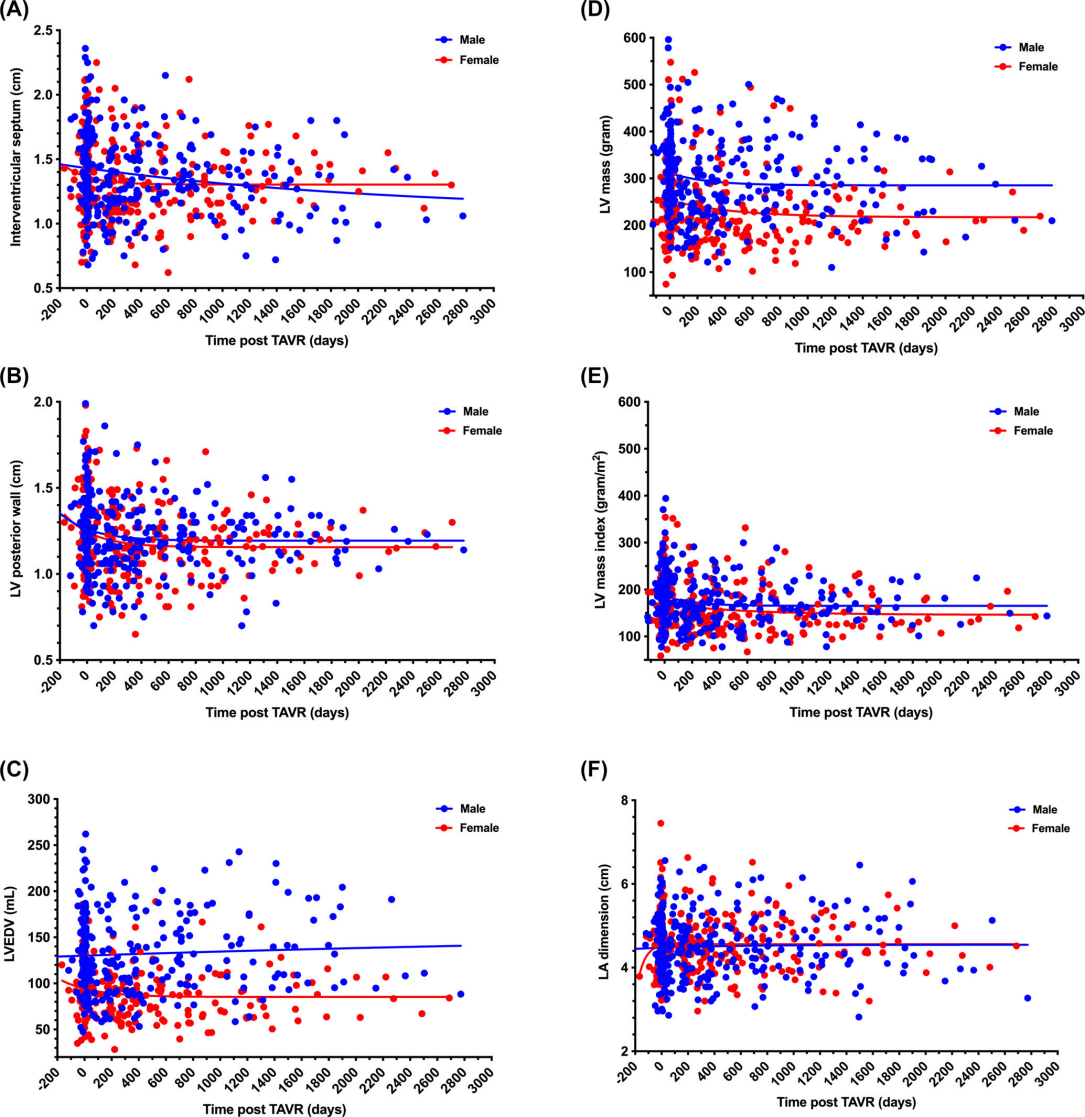
Changes in left ventricular and atrial size after transcatheter aortic valve replacement. All the calculated values are shown in scattered points with nonlinear regression curves. Blue points and lines for males; red points and lines for females. LA, left atrium; LV, left ventricle; LVEDV, left ventricle end‐diastole volume.

**FIGURE 3:**
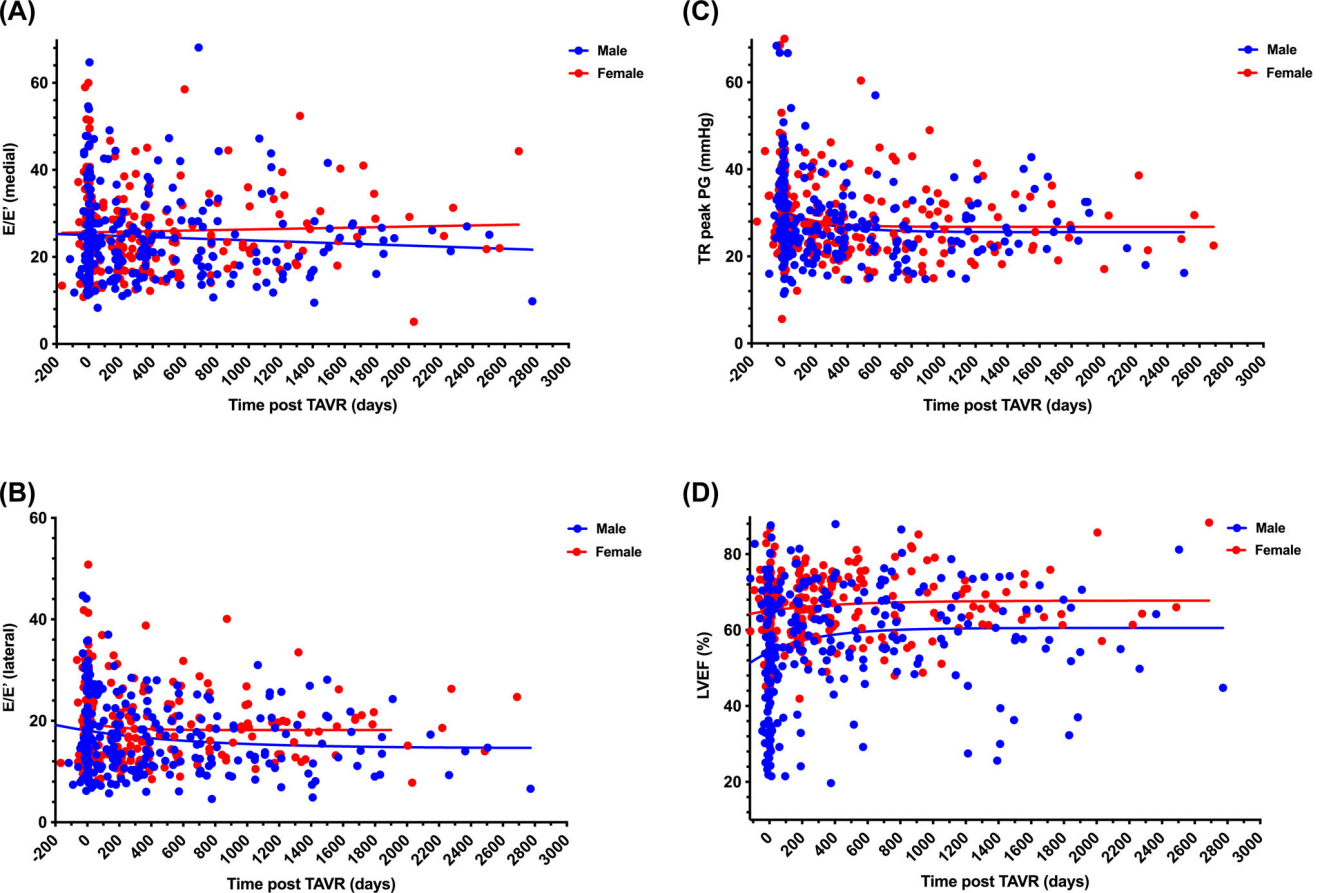
Changes in hemodynamic parameters after transcatheter aortic valve replacement. All the calculated values are shown in scattered points with nonlinear regression curves. Blue points and line for the male; red points and pink line for the female. LVEF, left ventricular ejection fraction; TR, tricuspid regurgitation; *E*/*E*′, ratio of mitral *E* flow velocity to tissue Doppler mitral ring velocity.

**FIGURE 4:**
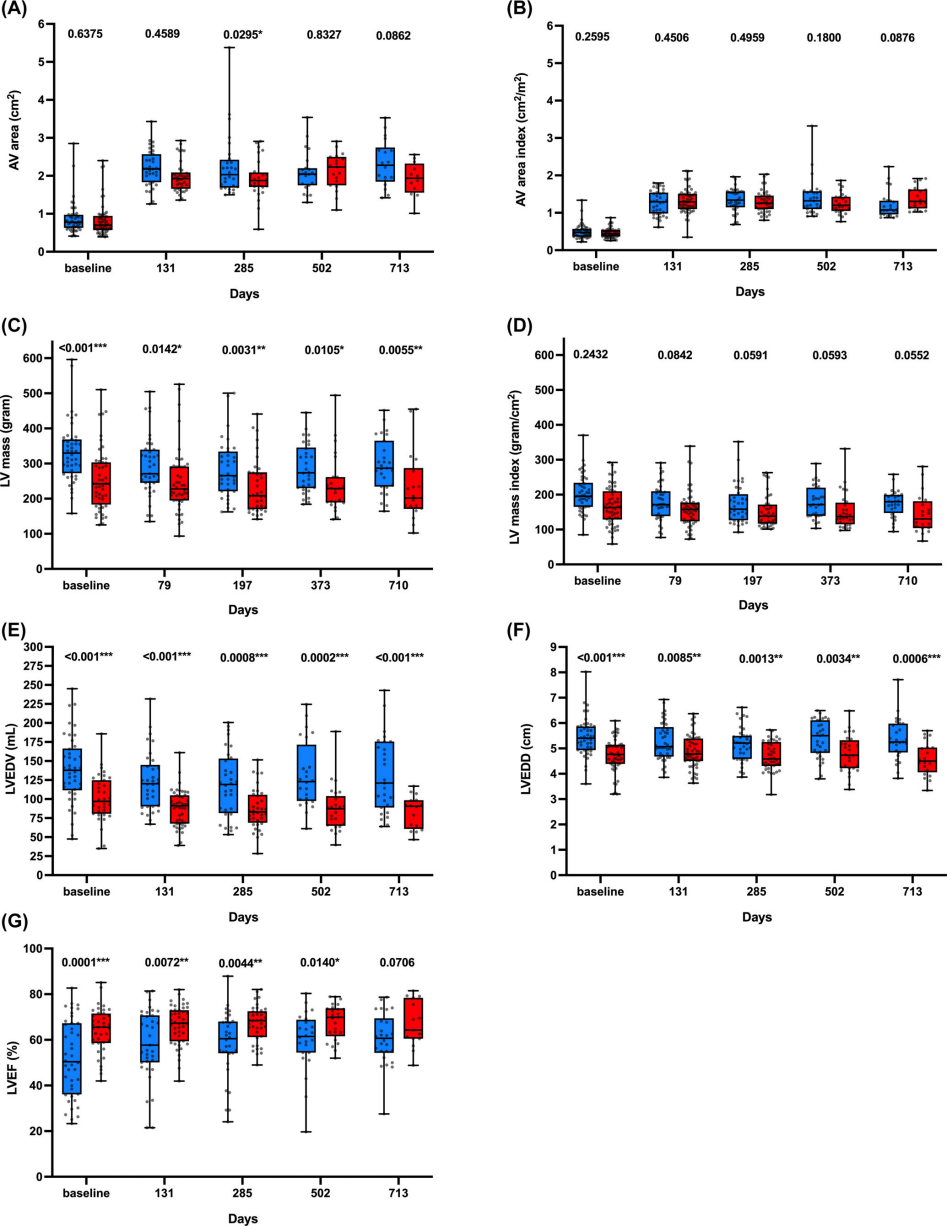
Changes in echocardiographic parameters from baseline to post‐TAVR follow‐up. Male in blue and female in red. The days indicate the post‐TAVR medium follow‐up time. The *p* values of each pair comparison are labeled at the top of all the histograms. AV, aortic valve; LVEDV, left ventricular end‐diastolic volume; LVEDD, left ventricular end‐diastolic diameter; LVEF, left ventricular ejection fraction.

All patients completed echocardiographic assessment within 3 months before the TAVR procedure as baseline data and before hospital discharge. The assessment and measurements of left ventricle (LV) such as interventricular septum (IVS), LV posterior wall thickness (LVPWd), LV end‐diastole dimension, and LV end‐diastole volume (LVEDV) were performed according to the standards of the American Society of Echocardiography.^9^ The left atrial diameter (LAD) was assessed using M‐mode in the parasternal longitudinal view. Left ventricular ejection fraction (LVEF) was assessed using the Simpson method in the apical 4‐chamber view. In addition, left ventricular mass (LV mass) was calculated using the Devereux‐modified method, and the LV mass index (LVMI) was calculated by dividing the LV mass by the body surface area. The degree of paravalvular leak after TAVR was classified using a semi‐quantitative method as follows: with the circumferential extent of paravalvular regurgitation, mild <10%, moderate 10%–29%, and severe ≥30%. According to VARC‐3, the study recorded the overall changes in echocardiographic parameters (Figure 1, 2, 3) and specified the outcomes at baseline (pre‐TAVR) and four time points of post‐TAVR follow‐up, as illustrated (Figure 4).

### 2.4 Procedure of TAVR

The standardized TAVR procedure was performed for all subjects using either a transfemoral or a transaortic approach under general anesthesia. The prosthetic valves adopted in the study were either self‐expandable (Medtronic CoreValve ReValving System; Medtronic, Minneapolis, MN, USA) or balloon‐expandable (Edwards SAPIEN valve; Edwards Lifesciences, Irvine, CA, USA).

### 2.5 Statistical analysis

The continuous variables at baseline were presented as arithmetic means ± SD and compared between sexes by independent *t*‐test, except for Logistic EuroSCORE I, STS score, AV mean PG, AV area (continuity equation VTI) [cm^2^], LVEF (Simpson's method) [%], which were analyzed using Mann–Whitney *U* tests because of non‐normal distribution. Statistical analysis was performed using Fisher's exact test, with a sample size less than five. Relationships between categorical variables and sex were described by counts and percentages and determined using chi‐square tests or Fisher's exact tests (i.e., comparisons of baseline characteristics, echocardiographic and procedural characteristics, and complications). Statistical significance was set at *p* < 0.05. We inspected the cardiac function parameters between sex over time and temporal trends using scatter plots and non‐linear fits. To assess the effect of sex on time‐changeable cardiac function of the echocardiologic parameters throughout the pre‐ and post‐TAVR periods, the generalized linear mixed model (GLMM), which is an extension of the generalized linear model (GLM), was adopted in the study using female sex as the reference/control group after considering time influences. We further applied box plots to describe the distribution of sex affecting cardiac function parameters during the post‐TAVR period. In addition, Kaplan–Meier survival analysis was used to estimate the cumulative survival proportion during post‐TAVR follow‐up. A two‐tailed *p*‐value of <0.05 was considered statistically significant. Data were analyzed using the IBM SPSS Statistics software version 20 (SPSS Inc., Chicago, IL, USA). Statistical graphs were generated using GraphPad Prism 9 Software (GraphPad Software, Boston, MA, USA).

## 3 RESULTS

### 3.1 Demographics

The baseline characteristics categorized by sex are summarized in Table 1. Among the 100 patients included, men (*n* = 46) and women (*n* = 54) had similar age (86.0 ± 6.4 years for men and 84.5 ± 5.8 years for women, respectively). Functional capacity was similar between sexes, with a comparable distribution of New York Heart Association (NYHA) class III/IV (*p* = 0.641). Regarding comorbidities, women had more hypertension than men did (men 71.7% vs. women 90.7%, *p* < 0.05). Men and women with diabetes mellitus, atrial fibrillation, chronic kidney disease (CKD), and the presence of an implanted pacemaker were comparable. Before TAVR, men had more coexisting moderate to severe AR. In addition, the coexistence of significant mitral regurgitation (MR) and tricuspid regurgitation (TR) was similar between sexes.

**TABLE 1:** Clinical and echocardiographic parameters prior to TAVR.

Parameters	All patients (*n* = 100)	Men (*n* = 46)	Women (*n* = 54)	*p*‐Value
Demographics, comorbidities, and clinical parameters				
Age	85.2 ± 6.1	86.0 ± 6.4	84.5 ± 5.8	0.2367
Height (m)	1.56 ± 0.08	1.62 ± 0.06	1.50 ± 0.06	0.3947
Weight (kg)	58.8 ± 11.5	61.9 ± 11.6	56.1 ± 10.7	0.3947
Body mass index (kg/m^2^)	24.2 ± 4.3	23.5 ± 3.9	24.8 ± 4.5	0.4012
NYHA class, *n* (%)				0.6405
III	54 (54.0)	26 (56.5)	28 (51.9)	
IV	46 (46.0)	20 (48.1)	26 (48.1)	
Hypertension, *n* (%)	82 (82.0)	33 (71.7)	49 (90.7)	<0.05*
Diabetes mellitus, *n* (%)	44 (44.0)	20 (43.5)	24 (44.4)	0.923
Atrial fibrillation, *n* (%)	23 (23.0)	11 (23.9)	12 (22.2)	0.8413
CKD stage 3–5, *n* (%)	41 (41.0)	22 (47.8)	19 (35.2)	0.2002
History of MI, *n* (%)	22 (22.0)	11 (23.9)	11 (20.4)	0.6700
Previous PCI, *n* (%)	33 (33.0)	16 (34.8)	17 (31.5)	0.7264
Previous CABG, *n* (%)	4 (4.0)	3 (6.5)	1 (1.9)	1.00^‡^
Previous stroke/TIA, *n* (%)	11 (11.0)	6 (13.0)	5 (9.3)	0.5467
Pacemaker, *n* (%)	5 (5.0)	1 (2.2)	4 (7.4)	0.2314
Logistic EuroSCORE I (%)	29.1 ± 12.8	29.2 ± 13.1	29.0 ± 12.7	0.9269
AR before				<0.05*
0	21 (21.0)	10 (22.2)	11 (21.6)	
1	38 (38.0)	11 (24.4)	27 (52.9)	
2	31 (31.0)	19 (42.2)	12 (23.5)	
3	6 (6.0)	5 (11.1)	1 (2.0)	
STS score (%)	17.4 ± 13.7	18.5 ± 14.6	16.6 ± 12.9	0.4928
Echocardiographic parameters				
AV mean PG [mmHg]	46.4 ± 17.8	47.5 ± 16.0	45.7 ± 19.2	0.0615
AV area (continuity equation VTI) [cm^2^]	0.8 ± 0.3	0.8 ± 0.3	0.7 ± 0.2	<0.05*
AV area index (cm^2^/m^2^)	0.5 ± 0.2	0.5 ± 0.1	0.5 ± 0.2	0.8226
LVEF (Simpson's method) [%]	57.1 ± 16.0	54.7 ± 17.2	59.6 ± 14.0	<0.01**
LV mass (g)	305.3 ± 87.2	323.9 ± 89.1	278.3 ± 78.1	<0.001***
LV mass index (g/m^2^)	180.5 ± 56.8	188.8 ± 60.0	175.2 ± 52.7	0.2432

*Note*: Statistical significance is presented as *p* < 0.05*, *p* < 0.01**, and *p* < 0.001***.

*Abbreviations*: AV, aortic valve; CABG, coronary artery bypass graft; CKD, chronic kidney disease; LVEF, left ventricle ejection fraction; MI, myocardial infarction; NYHA, New York Heart Association; PCI, percutaneous coronary intervention; PG, pressure gradient; STS, Society of Thoracic Surgeons; TIA, transient ischemic attack.

In addition, men and women had similar incidences of coronary heart disease, including old myocardial infarction, previous percutaneous coronary intervention (PCI), and coronary artery bypass graft surgery (CABG). The surgical risk scores, that is, the Logistic EuroSCORE I and STS scores, were similar.

All patients had severe AS prior to TAVR. The baseline aortic valve area, which was calculated using continuity equation in echocardiography, was smaller in women than in men (women 0.7 ± 0.2 cm^2^ vs. men 0.8 ± 0.3 cm^2^, *p* < 0.05). Sex difference for AV area was not observed with body size adjustment and the AV area index was similar between sex groups (women 0.5 ± 0.12 cm^2^/m^2^ vs. men 0.5 ± 0.2 cm^2^/m^2^, *p* = 0.8226). Likewise, there were no significant differences in the peak and mean pressure gradients of AV. Hypertrophic remodeling of the LV quantified by LV mass, was more severe in men than in women (LV mass, men 323.9 ± 89.1 g/m^2^ vs. women 278.3 ± 78.1 g/m^2^, *p* < 0.05). The sex differences disappeared after body size correction (g/m^2^). LV systolic function, which is reflected by the LVEF, was similar in men and women.

### 3.2 The devices of TAVR and the procedure‐related parameters

The devices used for TAVR and procedure‐related parameters are listed in Table 2. The prosthesis sizes were significantly smaller in women (men 28.0 ± 1.9 cm^2^ vs. women 25.0 ± 2.4 cm^2^, *p* < 0.001). The self‐expanding device was available later than the balloon‐expandable devices and made uneven use of these two types of TAVR devices. Post‐TAVR paravalvular leak and AR, which were more severe than moderate, occurred commonly in men and women. TAVR was successfully performed in all 100 patients with severe AS. The peak and mean pressure gradients declined (Figure 1A,B), and the AV areas, as well as the AV area index, increased (Figure 1C,D) after the TAVR procedures (see Figure S1). Major adverse cardiovascular events (MACE) showed no difference between sexes. Vascular complications were defined according to the VACR‐3 criteria did not differ between sexes (Table 2). Minor vascular complications occurred in only two women (3.7%). Pacemaker rates post‐TAVR also showed no differences.

**TABLE 2:** The device and procedures‐related parameters of TAVR.

Parameters	All patients (*n* = 100)	Men (*n* = 46)	Women (*n* = 54)	*p*‐Value
Self‐expanding valve (SEV), *n* (%)	75 (75.0)	36 (78.3)	39 (72.2)	0.3700
Balloon‐expandable valve (BEV), *n* (%)	25 (26.0)	10 (21.7)	15 (29.6)	0.4870
Second valve, *n* (%)	11 (11.0)	6 (13.0)	5 (9.3)	0.5467
SEV size (mm)	27.2 ± 2.3	26.0 ± 2.0	28.4 ± 1.8	<0.001***
BEV size (mm)	24.0 ± 2.3	22.6 ± 1.5	26.2 ± 1.4	<0.001***
AR or PVL after				0.6188
0	9 (9.0)	3 (6.8)	6 (11.3)	
1	71 (71.0)	33 (75.0)	38 (71.7)	
2	16 (16.0)	7 (15.9)	9 (17.0)	
3	1 (1.0)	1 (2.3)	0 (0.0)	
MR before				0.3470
0	14 (14.0)	4 (8.9)	10 (19.6)	
1	42 (42.0)	22 (48.9)	20 (39.2)	
2	36 (36.0)	18 (40.0)	18(35.3)	
3	4 (4.0)	1 (2.2)	3 (5.9)	
MR after				0.3578
0	2 (2.0)	0 (0.0)	2 (3.7)	
1	65 (65.0)	32 (72.7)	33 (61.1)	
2	30 (30.0)	12 (27.3)	18 (33.3)	
3	1 (1.0)	0 (0.0)	1 (1.9)	
Vascular complications, *n* (%)	6 (6.0)	3 (6.5)	3 (5.6)	0.2165
Major, *n* (%)	4 (4.0)	3 (6.5)	1(1.9)	
Minor, *n* (%)	2 (2.0)	0 (0.0)	2 (3.7)	
Pacemaker rates, *n* (%)	7 (7.0)	3 (6.5)	4 (7.4)	0.8626

*Note*: Statistical significance is presented as *p* < 0.05*, *p* < 0.01**, and *p* < 0.001***. Major vascular complications include acute cardiac tamponade with ECMO, acute stroke with right hemiplegia, and ascending aorta dissection. Minor vascular complications include right lower leg hematoma, and ecchymosis, and right forearm phlebitis.

*Abbreviations*: AR, aortic regurgitation; MR: mitral regurgitation; PVL, paravalvular leak.

### 3.3 Post‐TAVR regression of cardiac remodeling

The IVS, LVPW, LVEDV, LV mass, LV mass index, and LA diameter on echocardiography at baseline and during follow‐up are shown in Figure 2A–F to demonstrate changes in preexisting cardiac remodeling. The IVS and LVPW slightly decreased and later remained stable during the post‐TAVR follow‐up without sex‐related differences (Figure 2A,B). Regression of LV hypertrophy was shown with concordant reduction in LVEDV, LV mass, and LV mass index, which occurred mostly within post‐TAVR 200 days in both sex groups (Figure 2D,E). Interestingly, the reduction in LVEDV was greater in women (Figure 2C). For LA dilatation, there were no significant changes in the post‐TAVR follow‐up in either men or women (Figure 2F).

### 3.4 Changes of hemodynamic parameters and LV systolic function after TAVR

The hemodynamic parameters before and after TAVR are shown in Figure 3 and Table S1. The values of *E*/*E′*, which reflects LV filling pressure, declined after TAVR without statistical difference in both men and women, without sex differences (Figure 3A,B). Similarly, there was no sex difference in the TR peak pressure gradient, which reflects the pulmonary systolic pressure (Figure 3C). The improvement in LV systolic function was significant for both men and women, and women showed better improvement than men (Figure 3D).

### 3.5 Sex difference for changes of echocardiographic parameters from baseline to post‐TAVR follow‐up

To precisely understand sex differences in cardiac remodeling and functional changes during post‐TAVR follow‐up, a statistical method of mixed model analysis was conducted using women as the reference/control group. Table 3 presents the results. Notably, the mean AV area, LVEDV, and LVEDD were significantly larger in men (+0.1548 cm^2^, +39.77 mL, and +0.5983 cm for men, respectively; Table 3). In contrast, women showed significantly better improvement in LVEF than men (Table 3).

**TABLE 3:** Estimated values of echocardiologic parameters by mixed model.

	Estimate	95% CI lower	95% CI upper	*p*‐Value
AV area (cm^2^)				
Women	0 [reference]			
Men	0.15	0.02	0.29	<0.05*
AV area index (cm^2^/m^2^)				
Women	0 [reference]			
Men	0.01	0.02	0.29	0.8478
AV mean PG (mmHg)				
Women	0 [reference]			
Men	−2.3	−4.60	0.08	0.059
AV peak PG (mmHg)				
Women	0 [reference]			
Men	−3.56	−7.27	0.15	0.061
*E*/*E*′ (lat.)				
Women	0 [reference]			
Men	−3.56	−7.27	0.15	0.061
*E*/*E*′ (med.)				
Women	0 [reference]			
Men	−3.56	−7.27	0.15	0.061
LV mass (g)				
Women	0 [reference]			
Men	62.36	36.42	88.29	<0.001***
LV mass index (g/m^2^)				
Women	0 [reference]			
Men	25.79	41.61	9.98	<0.05*
LVEF (%)				
Women	0 [reference]			
Men	−8.83	−12.72	−4.93	<0.001***
IVS (cm)				
Women	0 [reference]			
Men	0.07	−0.02	0.15	0.137
LVPWd (cm)				
Women	0 [reference]			
Men	0.03	−0.03	0.09	0.329
LVEDV (mL)				
Women	0 [reference]			
Men	39.77 ± 6.08	27.85	51.69	<0.001***
LVEDD (cm)				
Women	0 [reference]			
Men	0.60	0.37	0.83	<0.001***
LAD (cm)				
Women	0 [reference]			
Men	−0.07	−0.31	0.17	0.588
TR peak PG (mmHg)				
Women	0 [reference]			
Men	−0.97	−3.47	1.53	0.448

*Note*: Statistical significance is presented as *p* < 0.05*, *p* < 0.01**, and *p* < 0.001***.

*Abbreviations*: AV, aortic valve area; IVS, interventricular septum; LAD, left atrial dimension; LVEDD, left ventricular end diastolic diameter; LVEDV, left ventricular end diastolic volume; LVPWd, left ventricular poster wall diameter; PG, pressure gradient; TRPPG, tricuspid valve regurgitation pressure gradient.

The alteration of specific parameters to demonstrate sex‐based differences at five time points was exhibited: baseline, 4 months, 1 year, one‐and‐half year, and 2 years (Figure 4). Men had larger AV area at baseline (men 0.8 cm^2^ vs. women 0.7 cm^2^, *p* < 0.05) and a larger post‐TAVR mean AV area (+0.1548 cm^2^ to women) (Figure 4A). With consideration of body size, men and women had similar AV area indices (men 0.5 ± 0.1 cm^2^/m^2^ vs. women = 0.5 ± 0.2 cm^2^/m^2^, *p* = 0.8226). As shown in Table 3 and Figure 4D, the post‐TAVR AV area showed no sex disparity (*p* = 0.8478). Regarding changes in LV remodeling, sex differences in LV mass, LVEDV, and LVEDD were observed (Figure 4B,C), suggesting inherited sex‐based differences in AS‐related LV remodeling and sex differences in post‐TAVR regression. In this study, women had better LVEF at baseline, and the advantage persisted during follow‐up with smaller statistical dispersion (Figure 4D).

### 3.6 Clinical outcomes

**FIGURE 5:**
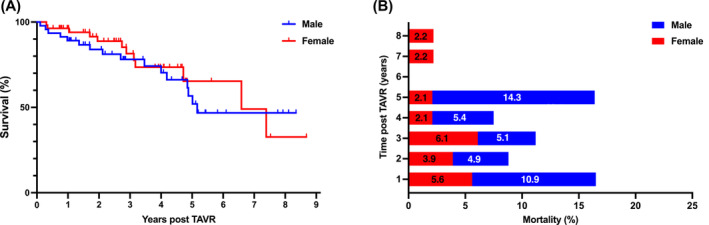
Clinical outcomes of 8.3 years in male and female TAVR patients. (A) Cumulative survival after TAVR. (B) Mortality in both sexes within each year. Male in blue and female in red.

The mortality rate was defined as the time from TAVR to death, and the entire follow‐up period was approximately 3000 days. The median follow‐up duration for the male and female groups was 986 and 909 days, respectively. There was no periprocedural mortality (within 30‐day) in either sex. As shown in Figure 5A, the cumulative 1‐, 2‐, and 5‐year survival rates were higher in women (94.0%, 88.8%, and 65.4%, respectively) than in men (89.1%, 83.9%, and 46.8%, respectively). Notably, the largest disparity occurred in the fifth year, as the survival rate of men dropped below 50%, whereas that of women remained above 50%. Figure 5B shows the mortality rates of both sexes at each post‐TAVR year. Women had a slightly higher mortality rate in the first, second, and third years, accounting for 5.6%, 3.9%, and 6.1%, respectively. In contrast, the mortality in the male group was distinctly higher in the first and fifth years (10.9% and 14.3%, respectively). The present study reported that not only the cumulative survival rate, but also the yearly mortality pattern post‐TAVR could exhibit sex‐based disparities.

## 4 DISCUSSION

The main findings of this study were as follows: (1) female patients exhibited more favorable post‐TAVR improvement of LV remodeling with better regression of LV mass, (2) women had better pre‐ and post‐TAVR LVEF values, and (3) women tended to have better survival outcomes after TAVR.

Regression of LVMI is inevitably observed in almost all post‐TAVR patients and it has been shown by previous studies that the LV mass at post‐TAVR 30 days is a significant predictor for a lower hospitalization for heart failure.^10^ Chen et al. reported that in severe AS, females had greater and earlier LV mass regression at 3 months after the surgery compared to male patients.^11^ In addition, the sex‐difference in AS‐related LV hypertrophy phenotype has been noticed in women with more concentric hypertrophy and men have more eccentric remodeling.^12^, ^13^ Our present study showed that the post‐TAVR sex‐difference in LV mass was persistent during follow‐up, suggesting that there are inherited sex differences in the regulation of LV hypertrophy while adapting pressure overload and the therapeutic unloading, particularly in cases of severe AS receiving TAVR or SAVR.^14^, ^15^

Regarding the better post‐TAVR LVEF in women, the underlying mechanisms have been elaborated and suggested with sex‐based hormonal and transcriptomic differences.^13^ Kararigas et al. reported that men have more fibrosis and collagen deposition in the overloaded ventricle, which manifests as activated fibrosis‐related genes/pathways.^14^ In addition, men have more late gadolinium enhancement (LGE) of the hypertrophic ventricle in cardiac magnetic resonance imaging, which is a marker of focal fibrosis and is related to adverse prognosis following AVR.^16^, ^17^ Better LV systolic function in women has been suggested due to lower end‐systolic wall stress compared with men,^18^, ^19^ and better LVEF affects mortality in post‐TAVR cases for both men and women.^17^, ^20^, ^21^ Another important outcome factor is the severity of paravalvular leak, which is commonly milder with smaller annular size in women.^22^ Although in the present study, women had unfavorable higher coexistence of hypertension,^23^ the outcome data revealed a trend for women with better survival during follow‐up.

The present study reported post‐TAVR mortality over 3000 days along with changes in echocardiographic parameters. Unsurprisingly, non‐cardiac late‐life morbidities affect overall post‐TAVR mortality. In the yearly mortality analysis, our study showed a higher mortality rate in men in the first post‐TAVR year (men, 10.9% vs. women, 5.6%; *p* = 0.3680). In another study, women had better short‐term (30‐day or in‐hospital) survival with a median follow‐up of approximately 1 year.^24^ Non‐cardiac causes of mortality such as sepsis and liver failure, as shown in Table S3 blunted the advantages of favorable LVEF in women for subsequent prognosis. In addition, women experienced more strokes, major vascular complications, and major bleeding events.^24^, ^25^ With multifactorial influence, the favorable post‐TAVR regress of LV hypertrophy was therefore not associated with less mortality.^26^, ^27^

This study had several limitations that need to be addressed. First, this study reported the first 100 cases that had been performed in a University Hospital before the TAVR was covered by the National Health Insurance reimbursement, which was started in April 2021. Therefore, the study population may have been biased by the limited number of cases with relatively high socioeconomic status. Second, the devices used in this study were either the Sapien or CoreValve prostheses. Therefore, these results may not be applicable to other devices or new‐generation technologies. Lastly, early and late rehospital readmissions that affect post‐TAVR mortality were not recorded in this study.^28^, ^29^

Our study suggests that sex‐related differences exist after TAVR by measuring echocardiographic parameters. Women had better LVEF and LVMI than men. The clinical outcome demonstrated that there were no sex differences but a favorable overall survival of women. Collectively, TAVR is a reliable procedure with satisfactory outcomes.

## CONFLICT OF INTEREST STATEMENT

All authors declare no conflict of interest.

## Supporting information


**TABLE S1.** Outcome of echocardiographic parameters in post‐TAVR follow‐up.
**TABLE S2.** Calculated values of echocardiologic parameters in follow‐up.
**TABLE S3.** Causes of mortality of post‐TAVR patients.

## References

[1] Aluru JS , Barsouk A , Saginala K , Rawla P , Barsouk A . Valvular heart disease epidemiology. Med Sci (Basel). 2022;10(2):32. 35736352 10.3390/medsci10020032 PMC9228968

[2] Marquis‐Gravel G , Redfors B , Leon MB , Genereux P . Medical treatment of aortic stenosis. Circulation. 2016;134(22):1766–1784. 27895025 10.1161/CIRCULATIONAHA.116.023997

[3] Leon MB , Smith CR , Mack M , Miller DC , Moses JW , Svensson LG , et al. Transcatheter aortic‐valve implantation for aortic stenosis in patients who cannot undergo surgery. N Engl J Med. 2010;363(17):1597–1607. 20961243 10.1056/NEJMoa1008232

[4] Smith CR , Leon MB , Mack MJ , Miller DC , Moses JW , Svensson LG , et al. Transcatheter versus surgical aortic‐valve replacement in high‐risk patients. N Engl J Med. 2011;364(23):2187–2198. 21639811 10.1056/NEJMoa1103510

[5] Vlastra W , Chandrasekhar J , Garcia Del Blanco B , Tchetche D , de Brito FS Jr , Barbanti M , et al. Sex differences in transfemoral transcatheter aortic valve replacement. J Am Coll Cardiol. 2019;74(22):2758–2767. 31562908 10.1016/j.jacc.2019.09.015

[6] Chiam PTL , Hayashida K , Watanabe Y , Yin WH , Kao HL , Lee MKY , et al. Sex differences in patients undergoing transcatheter aortic valve replacement in Asia. Open Heart. 2021;8(1):e001541. 33419935 10.1136/openhrt-2020-001541 PMC7798412

[7] Pighi M , Piazza N , Martucci G , Lachapelle K , Perrault LP , Asgar AW , et al. Sex‐specific determinants of outcomes after transcatheter aortic valve replacement. Circ Cardiovasc Qual Outcomes. 2019;12(3):e005363. 30879326 10.1161/CIRCOUTCOMES.118.005363

[8] Valve Academic Research Consortium 3: Updated Endpoint Definitions for Aortic Valve Clinical Research. *J Am Coll Cardiol* . 2021;77(21):2717–2746. 10.1016/j.jacc.2021.02.038 33888385

[9] Lang RM , Badano LP , Mor‐Avi V , Afilalo J , Armstrong A , Ernande L , et al. Recommendations for cardiac chamber quantification by echocardiography in adults: an update from the American Society of Echocardiography and the European Association of Cardiovascular Imaging. J Am Soc Echocardiogr. 2015;28(1):1–39.e14. 25559473 10.1016/j.echo.2014.10.003

[10] Lindman BR , Stewart WJ , Pibarot P , Hahn RT , Otto CM , Xu K , et al. Early regression of severe left ventricular hypertrophy after transcatheter aortic valve replacement is associated with decreased hospitalizations. JACC Cardiovasc Interv. 2014;7(6):662–673. 24947722 10.1016/j.jcin.2014.02.011 PMC4165852

[11] Chen SC , Leu HB , Chang HH , Chen IM , Chen PL , Lin SM , et al. Women had favourable reverse left ventricle remodelling after TAVR. Eur J Clin Invest. 2020;50(1):e13183. 31691961 10.1111/eci.13183 PMC7050508

[12] Villari B , Campbell SE , Schneider J , Vassalli G , Chiariello M , Hess OM . Sex‐dependent differences in left ventricular function and structure in chronic pressure overload. Eur Heart J. 1995;16(10):1410–1419. 8746910 10.1093/oxfordjournals.eurheartj.a060749

[13] Petrov G , Regitz‐Zagrosek V , Lehmkuhl E , Krabatsch T , Dunkel A , Dandel M , et al. Regression of myocardial hypertrophy after aortic valve replacement: faster in women? Circulation. 2010;122(11 Suppl):S23–S28. 20837918 10.1161/CIRCULATIONAHA.109.927764

[14] Kararigas G , Dworatzek E , Petrov G , Summer H , Schulze TM , Baczko I , et al. Sex‐dependent regulation of fibrosis and inflammation in human left ventricular remodelling under pressure overload. Eur J Heart Fail. 2014;16(11):1160–1167. 25287281 10.1002/ejhf.171

[15] Goel H , Kumar A , Garg N , Mills JD . Men are from mars, women are from venus: factors responsible for gender differences in outcomes after surgical and trans‐catheter aortic valve replacement. Trends Cardiovasc Med. 2021;31(1):34–46. 31902553 10.1016/j.tcm.2019.11.010

[16] Weidemann F , Herrmann S , Stork S , Niemann M , Frantz S , Lange V , et al. Impact of myocardial fibrosis in patients with symptomatic severe aortic stenosis. Circulation. 2009;120(7):577–584. 19652094 10.1161/CIRCULATIONAHA.108.847772

[17] Barone‐Rochette G , Pierard S , Meester D , de Ravenstein C , Seldrum S , Melchior J , et al. Prognostic significance of LGE by CMR in aortic stenosis patients undergoing valve replacement. J Am Coll Cardiol. 2014;64(2):144–154. 25011718 10.1016/j.jacc.2014.02.612

[18] Douglas PS , Otto CM , Mickel MC , Labovitz A , Reid CL , Davis KB . Gender differences in left ventricle geometry and function in patients undergoing balloon dilatation of the aortic valve for isolated aortic stenosis. NHLBI Balloon Valvuloplasty Registry. Br Heart J. 1995;73(6):548–554. 7626355 10.1136/hrt.73.6.548 PMC483918

[19] Carroll JD , Carroll EP , Feldman T , Ward DM , Lang RM , McGaughey D , et al. Sex‐associated differences in left ventricular function in aortic stenosis of the elderly. Circulation. 1992;86(4):1099–1107. 1394918 10.1161/01.cir.86.4.1099

[20] Singh A , Musa TA , Treibel TA , Vassiliou VS , Captur G , Chin C , et al. Sex differences in left ventricular remodelling, myocardial fibrosis and mortality after aortic valve replacement. Heart. 2019;105(23):1818–1824. 31467152 10.1136/heartjnl-2019-314987 PMC6900227

[21] Naoum C , Blanke P , Dvir D , Pibarot P , Humphries K , Webb J , et al. Clinical outcomes and imaging findings in women undergoing TAVR. JACC Cardiovasc Imaging. 2016;9(4):483–493. 27056166 10.1016/j.jcmg.2016.02.009

[22] Saad M , Nairooz R , Pothineni NVK , Almomani A , Kovelamudi S , Sardar P , et al. Long‐term outcomes with transcatheter aortic valve replacement in women compared with men: evidence from a meta‐analysis. JACC Cardiovasc Interv. 2018;11(1):24–35. 29055767 10.1016/j.jcin.2017.08.015

[23] Gerdts E , Midtbø H . Time to integrate sex in management of aortic valve stenosis. Eur Cardiol. 2023;18:e05. 37456772 10.15420/ecr.2022.52 PMC10345976

[24] O'Connor SA , Morice MC , Gilard M , Leon MB , Webb JG , Dvir D , et al. Revisiting sex equality with transcatheter aortic valve replacement outcomes: a collaborative, patient‐level meta‐analysis of 11,310 patients. J Am Coll Cardiol. 2015;66(3):221–228. 26184614 10.1016/j.jacc.2015.05.024

[25] Conrotto F , D'Ascenzo F , Presbitero P , Humphries KH , Webb JG , O'Connor SA , et al. Effect of gender after transcatheter aortic valve implantation: a meta‐analysis. Ann Thorac Surg. 2015;99(3):809–816. 25633460 10.1016/j.athoracsur.2014.09.089

[26] Minamino‐Muta E , Kato T , Morimoto T , Taniguchi T , Inoko M , Haruna T , et al. Impact of the left ventricular mass index on the outcomes of severe aortic stenosis. Heart. 2017;103(24):1992–1999. 28684442 10.1136/heartjnl-2016-311022 PMC5749367

[27] Truong VT , Mazur W , Broderick J , Egnaczyk GF , Kereiakes DJ , Sarembock IJ , et al. Transcatheter aortic valve replacement and left ventricular geometry: survival and gender differences. J Am Soc Echocardiogr. 2020;33(11):1357–1362.e2. 32828622 10.1016/j.echo.2020.06.015

[28] Goldsweig A , Aronow HD . Identifying patients likely to be readmitted after transcatheter aortic valve replacement. Heart. 2020;106(4):256–260. 31649048 10.1136/heartjnl-2019-315381

[29] Nombela‐Franco L , del Trigo M , Morrison‐Polo G , Veiga G , Jimenez‐Quevedo P , Abdul‐Jawad Altisent O , et al. Incidence, causes, and predictors of early (</=30 days) and late unplanned hospital readmissions after transcatheter aortic valve replacement. JACC Cardiovasc Interv. 2015;8(13):1748–1757. 26476610 10.1016/j.jcin.2015.07.022

