# Supplementary material for: Female showed favorable left ventricle hypertrophy regression during post‐TAVR follow‐up
Source: Kaohsiung J Med Sci. 2024 Feb 8;40(4):384–94. doi: 10.1002/kjm2.12808 (PMC11895601; doi:10.1002/kjm2.12808)
Supplement: Supplementary file 1 — TABLE S1. Outcome of echocardiographic parameters in post‐TAVR follow‐up. TABLE S2. Calculated values of echocardiologic parameters in follow‐up. TABLE S3. Causes of mortality of post‐TAVR patients. [file KJM2-40-384-s001.docx]

**Supplementary Materials**

**Suppl. Table 1. Outcome of echocardiographic parameters in post-TAVR follow-up**

|  | Gender | | Baseline | | 1 year | | 2 years | | 3 years | 4 years | | 5 years | | Overall outcome | |
| --- | --- | --- | --- | --- | --- | --- | --- | --- | --- | --- | --- | --- | --- | --- | --- |
| AV area, cm^2^ | Men | | 0.8 ± 0.3 | | 2.1 ± 0.6 | | 2.2 ± 0.6 | | 2.2 ± 0.6 | 2.2 ± 0.6 | | 2.2 ± 0.6 | | 2.2 ± 0.6 | |
|  | Women | | 0.7 ± 0.2 | | 2.0 ± 0.5 | | 2.0 ± 0.5 | | 2.0 ± 0.5 | 2.0 ± 0.5 | | 2.0 ± 0.5 | | 2.0 ± 0.5 | |
| AV area index, cm^2^ / m^2^ | Men | 0.5 ± 0.1 | | 1.4 ± 0.4 | | 1.3 ± 0.4 | | 1.3 ± 0.4 | | | 1.3 ± 0.4 | | 1.3 ± 0.4 | | 1.3 ± 0.4 |
|  | Women | 0.5 ± 0.2 | | 1.3 ± 0.3 | | 1.3 ± 0.3 | | 1.3 ± 0.3 | | | 1.3 ± 0.3 | | 1.3 ± 0.3 | | 1.3 ± 0.3 |
| AV mean PG, mmHg | Men | | 75.1 ± 26.0 | | 17.5 ± 7.0 | | 17.5 ± 7.1 | | 17.7 ± 7.1 | 17.6 ± 7.2 | | 17.6 ± 7.1 | | 17.3 ± 7.1 | |
|  | Women | | 82.4 ± 28.4 | | 20.6 ± 9.9 | | 21.1 ± 9.8 | | 20.5 ± 9.7 | 20.0 ± 9.7 | | 19.9 ± 9.6 | | 19.8 ± 9.5 | |
| AV peak PG, mmHg | Men | | 75.1 ± 26.0 | | 17.5 ± 7.0 | | 17.5 ± 7.1 | | 17.7 ± 7.1 | 17.6 ± 7.2 | | 17.6 ± 7.1 | | 17.3 ± 7.1 | |
|  | Women | | 82.4 ± 28.4 | | 20.6 ± 9.9 | | 21.1 ± 9.8 | | 20.5 ± 9.7 | 20.0 ± 9.7 | | 19.9 ± 9.6 | | 19.8 ± 9.5 | |
| LVEF, % | Men | | 51.1 ± 17.4 | | 57.1 ± 14.8 | | 57.5 ± 14.5 | | 58.3 ± 14.1 | 58.5 ± 14.2 | | 58.5 ± 14.1 | | 58.3 ± 14.1 | |
|  | Women | | 64.4 ± 9.9 | | 65.6 ± 8.9 | | 66.2 ± 8.6 | | 66.2 ± 9.1 | 66.2 ± 8.8 | | 66.3 ± 8.7 | | 66.4 ± 8.8 | |
| IVS, cm | Men | | 1.5 ± 0.3 | | 1.4 ± 0.4 | | 1.4 ± 0.3 | | 1.4 ± 0.3 | 1.4 ± 0.3 | | 1.4 ± 0.3 | | 1.4 ± 0.3 | |
|  | Women | | 1.4 ± 0.3 | | 1.3 ± 0.3 | | 1.3 ± 0.3 | | 1.3 ± 0.3 | 1.3 ± 0.3 | | 1.3 ± 0.3 | | 1.3 ± 0.3 | |
| LAD, cm | Men | | 4.6 ± 0.7 | | 4.5 ± 0.8 | | 4.5 ± 0.7 | | 4.5 ± 0.7 | 4.5 ± 0.7 | | 4.5 ± 0.8 | | 4.5 ± 0.8 | |
|  | Women | | 4.5 ± 0.9 | | 4.5 ± 0.7 | | 4.5 ± 0.7 | | 4.6 ± 0.7 | 4.5 ± 0.7 | | 4.5 ± 0.7 | | 4.5 ± 0.7 | |
| LVEDV, mL | Men | | 140.1 ± 43.3 | | 125.8 ± 42.2 | | 126.1 ± 42.2 | | 128.3 ± 41.9 | 128.5 ± 42.7 | | 130.0 ± 43.3 | | 130.3 ± 43.1 | |
|  | Women | | 98.6 ± 30.0 | | 89.4 ± 26.9 | | 90.2 ± 27.5 | | 89.2 ± 27.8 | 88.6 ± 27.8 | | 88.8 ± 27.6 | | 88.7 ± 27.3 | |
| TR peak PG, mmHg | Men | | 34.7 ± 11.5 | | 27.2 ± 9.2 | | 27.2 ± 8.9 | | 26.7 ± 8.6 | 26.6 ± 8.4 | | 26.8 ± 8.4 | | 26.7 ± 8.3 | |
|  | Women | | 32.5 ± 11.0 | | 28.7 ± 8.3 | | 28.4 ± 8.6 | | 28.2 ± 8.7 | 28.0 ± 8.5 | | 28.0 ± 8.4 | | 28.0 ± 8.4 | |
| LVPWd, cm | Men | | 1.3 ± 0.2 | | 1.2 ± 0.2 | | 1.2 ± 0.2 | | 1.2 ± 0.2 | 1.2 ± 0.2 | | 1.2 ± 0.2 | | 1.2 ± 0.2 | |
|  | Women | | 1.2 ± 0.3 | | 1.2 ± 0.2 | | 1.2 ± 0.2 | | 1.2 ± 0.2 | 1.2 ± 0.2 | | 1.2 ± 0.2 | | 1.2 ± 0.2 | |
| LV mass, g | Men | | 330.5 ± 85.7 | | 292.8 ± 85.4 | | 293.5 ± 85.5 | | 297.1 ± 84.7 | 295.8 ± 84.3 | | 295.6 ± 83.6 | | 293.5 ± 83.4 | |
|  | Women | | 251.0 ± 91.1 | | 247.7 ± 87.4 | | 242.3 ± 86.2 | | 240.8 ± 86.0 | 238.8 ± 83.3 | | 238.5 ± 82.4 | | 237.6 ± 81.4 | |
| LV mass index, g/m^2^ | Men | 190.2 ± 60.2 | | 177.1 ± 52.1 | | 175.1 ± 51.8 | | 175.2 ± 51.3 | | | 174.0 ± 50.2 | | 173.8 ± 49.6 | | 173.4 ± 49.4 |
|  | Women | 171.4 ± 52.3 | | 164.0 ± 53.5 | | 161.6 ± 53.6 | | 161.4 ± 53.5 | | | 160.2 ± 52.6 | | 159.9 ± 52.0 | | 159.0 ± 51.1 |
| E/E’ (lat) | Men | | 18.2 ± 9.7 | | 17.5 ± 7.6 | | 17.3 ± 7.2 | | 17.0 ± 7.1 | 17.0 ± 7.0 | | 16.8 ± 7.0 | | 16.7 ± 6.9 | |
|  | Women | | 19.7 ± 8.2 | | 18.8 ± 7.8 | | 18.7 ± 7.6 | | 18.9 ± 7.6 | 18.7 ± 7.4 | | 18.8 ± 7.3 | | 18.8 ± 7.2 | |
| E/E’ (med) | Men | | 25.0 ± 12.1 | | 24.3 ± 10.0 | | 25.1 ± 10.4 | | 24.6 ± 10.2 | 24.5 ± 10.2 | | 24.5 ± 10.1 | | 24.4 ± 9.9 | |
|  | Women | | 27.6 ± 12.2 | | 25.3 ± 8.8 | | 25.0 ± 9.0 | | 25.3 ± 9.6 | 25.4 ± 9.5 | | 25.5 ± 9.5 | | 25.6 ± 9.6 | |

Variables presented as mean ± standard deviation. AV, aortic valve area; PG, pressure gradient; IVS, interventricular septum; LVEF, left ventricle ejection fraction; LVPWd, left ventricular poster wall diameter; LAD, left atrial dimension; LVEDV, left ventricular end diastolic volume; TRPPG, tricuspid valve regurgitation pressure gradient

**Suppl. Table 2. Calculated values of echocardiologic parameters in follow-up**

|  | Sex | Baseline | 4 months | 1 year | 1.5 year | 2 years | Estimate^†^ | p-value^†^ |
| --- | --- | --- | --- | --- | --- | --- | --- | --- |
| AV area, cm^2^ | Men | 0.9 ± 0.4 | 2.1 ± 0.5 | 2.2 ± 0.5 | 2.3 ± 0.8 | 2.1 ± 0.5 | 0.15 | 0.0285 |
|  | Women | 0.8 ± 0.4 | 2.0 ± 0.5 | 2.0 ± 0.4 | 1.9 ± 0.5 | 2.1 ± 0.5 |  |  |
| LV mass, g/m^2^ | Men | 332.5 ± 86.1 | 292.0 ± 82.7 | 286.1 ± 80.8 | 290.5 ± 72.7 | 301.4 ± 81.5 |  |  |
|  | Women | 254.5 ± 88.4 | 246.7 ± 90.3 | 230.8 ± 77.5 | 238.5 ± 77.6 | 226.8 ± 92.7 |  |  |
| LVEDV, mL | Men | 138.9 ± 44.6 | 123.5 ± 38.7 | 119.0 ± 43.7 | 131.4 ± 43.8 | 131.3 ± 49.6 | 39.77 | <.0001 |
|  | women | 99.6 ± 30.2 | 88.1 ± 23.9 | 87.0 ± 26.9 | 87.4 ± 30.6 | 82.9 ± 21.7 |  |  |
| LVEF, % | Men | 51.8 ± 16.9 | 58.3 ± 15.5 | 58.3 ± 14.6 | 59.8 ± 13.3 | 60.4 ± 11.4 | -8.83 | <.0001 |
|  | women | 64.2 ± 9.8 | 66.5 ± 8.8 | 66.9 ± 8.1 | 67.9 ± 7.9 | 66.9 ± 10.1 |  |  |
| LVEDD, cm | Men | 5.5 ± 0.8 | 5.3 ± 0.8 | 5.2 ± 0.7 | 5.4 ± 0.8 | 5.4 ± 0.8 | 0.60 | <.0001 |
|  | women | 4.8 ± 0.7 | 4.9 ± 0.7 | 4.7 ± 0.6 | 4.8 ± 0.7 | 4.6 ± 0.7 |  |  |
| ^†^The estimated value and p-values were calculated by mixed models using female as reference. AV, aortic valve area; LVEDV, left ventricular end diastolic volume; LVEDD, left ventricular end diastolic diameter | | | | | | | | |

**Suppl. Table 3. Causes of mortality of post-TAVR patients**

|  | Total  (n= 100) | Men  (n= 46) | Women  (n= 54) |
| --- | --- | --- | --- |
| All-cause mortality | 28 (28.0) | 16 (34.8) | 12 (22.2) |
| Cardiac mortality | 6 (21.4) | 3 (18.8) | 3 (25.0) |
| Heart failure | 1 (3.6) | 1 (6.3) | 0 (0.0) |
| NSTEMI | 1 (3.6) | 1 (6.3) | 0 (0.0) |
| STEMI | 1 (3.6) | 0 (0.0) | 1 (8.3) |
| Acute aortic dissection | 1 (3.6) | 0 (0.0) | 1 (8.3) |
| ER OHCA | 2 (7.1) | 1 (6.3) | 1 (8.3) |
| Non-cardiac mortality | 22 (78.6) | 13 (81.3) | 9 (16.7) |
| Hypokalemia | 1 (3.6) | 0 (0.0) | 1 (8.3) |
| Sepsis^†^ | 6 (21.4) | 3 (18.8) | 3 (25.0) |
| Liver failure | 1 (3.6) | 1 (6.3) | 0 (0.0) |
| Infection | 1 (3.6) | 1 (6.3) | 0 (0.0) |
| Hyperkalemia | 1 (3.6) | 0 (0.0) | 1 (8.3) |
| Conscious coma | 1 (3.6) | 1 (6.3) | 0 (0.0) |
| Pneumonia | 3 (10.7) | 3 (18.8) | 0 (0.0) |
| Cancer | 1 (3.6) | 0 (0.0) | 1 (8.3) |
| Others or undetermined | 7 (25.0) | 4 (25.0) | 3 (25.0) |

^†^Septic shock is included in sepsis.
